# Biofilms on Plastic Debris and the Microbiome

**DOI:** 10.3390/microorganisms12071362

**Published:** 2024-07-02

**Authors:** Yiqian Qian, Lingfeng Huang, Pei Yan, Xinhong Wang, Yuanrong Luo

**Affiliations:** 1 Key Laboratory of the Ministry of Education for Coastal and Wetland Ecosystems, Xiamen University, Xiamen 361102, China; 2 Xiamen City Key Laboratory of Urban Sea Ecological Conservation and Restoration (USER), Xiamen University, Xiamen 361102, China; 3 College of the Environment and Ecology, Xiamen University, Xiamen 361102, China; 4 State Key Laboratory of Marine Environmental Science, Xiamen University, Xiamen 361102, China

**Keywords:** plastic debris, microbiome, metagenome, biofilm

## Abstract

Plastic pollution has become a global environmental problem, and the large number of microorganisms attached to plastic debris in the environment has become a hot topic due to their rapid response to pollutants and environmental changes. In this study, we used high-throughput sequencing to investigate the microbial community structure of and explore the metagenome in the biofilm of two types of plastic debris, polystyrene (PS) and polyethylene terephthalate (PET), and compared them with a water sample collected at the sampling site. The phylum Proteobacteria dominated both the PET and PS samples, at 93.43% and 65.95%, respectively. The metagenome data indicated that the biofilm is enriched with a number of hydrocarbon (petroleum, microplastics, etc.) degrading genes. Our results show that the type of plastic determined the bacterial community structure of the biofilm, while the environment had relatively little effect.

## 1. Introduction

Plastic production has been growing exponentially due to the immense global demand, making plastic pollution one of the most pressing environmental issues. Plastics constitute the largest, most harmful, and most persistent fraction of marine litter, accounting for at least 85 percent of the total marine waste [1]. The widespread disposal of plastic is causing severe environmental problems worldwide. Plastic debris floating on the ocean can act as a carrier for heavy metals, organic pollutants, and antibiotics [2,3]. Moreover, it serves as a novel artificial interface, providing substrates and habitats for microorganisms to attach to and form biofilms, which are commonly referred to as the plastisphere [4].

Recent studies have revealed that the plastisphere harbors a diverse microbial community, which is distinct from the surrounding seawater [5]. The biofilm formation on plastic debris is a complex process that involves the initial attachment of pioneer microorganisms, followed by the recruitment of secondary colonizers [6]. As the biofilm matures, it develops into a highly organized structure with distinct microbial populations occupying different niches [7]. The enrichment of microorganisms involved in the degradation and transformation of contaminants, as well as those participating in the biogeochemical cycles of carbon, nitrogen, and other elements, contributes to the rich microbial diversity observed in the plastisphere [4].

Advances in molecular techniques, such as high-throughput sequencing, have greatly enhanced our understanding of the bacterial community structure and microbiome associated with plastic debris biofilms. Oberbeckmann et al. used Illumina sequencing to compare the microbial communities on polyethylene terephthalate (PET) bottles and glass slides in the North Sea [5]. They observed that the bacterial community structure on PET bottles was significantly different from that on glass slides, with a higher abundance of Cyanobacteria and a lower abundance of Gammaproteobacteria on the plastic substrates. Debroas et al. used metagenomic sequencing to investigate the microbial communities on polyethylene plastic bags collected from the North Atlantic Subtropical Gyre [8]. They found that the plastic biofilms were enriched in bacterial genera known to degrade hydrocarbons, such as *Alcanivorax*, *Marinobacter*, and *Aestuariibacter*, suggesting that these bacteria may play a role in the degradation of plastic polymers. Furthermore, the plastisphere has been found to harbor antibiotic resistance genes and potentially pathogenic bacteria, raising concerns about the role of plastic debris in the dissemination of these threats [9]. Amaral-Zettler et al. used 16S rRNA gene sequencing to investigate the effects of geography and plastic type on the microbial communities of marine plastic debris [10]. They found that the bacterial community structure varied significantly between different geographic locations and plastic types, with polypropylene and polyethylene harboring distinct microbial communities compared to polystyrene and polyethylene terephthalate. These studies have revealed the diverse and dynamic nature of these biofilms, identified key bacterial taxa involved in plastic degradation, and highlighted the potential role of plastic debris in the spread of antibiotic resistance and pathogenic bacteria.

Despite the growing interest in plastisphere ecology, little is known about how microorganisms interact with synthetic polymers in the environment. To explore plastisphere ecology, we collected two types of plastic debris, foam (polystyrene, PS) and bottles (polyethylene terephthalate, PET), floating on coast seawater surfaces and investigated the microbial community structure of the biofilms and functional genome. Our study aims to provide insights into the ecological impact of the plastisphere and contributes to the understanding of the complex interactions between microorganisms and plastic debris in marine environments.

## 2. Materials and Methods

### 2.1. Sample Collection

In August 2021, two types of floating plastic debris, foam (PS) and bottles (PET), were collected on the Dongshan Bay coast (117°31′18″, 23°44′38″). Each type of plastic consisted of 3 samples and was collected at the same place. A total of 1 L of surface seawater (Water) at the sampling site was also collected. There is a catering industry near the sampling site. After the samples were collected on site, they were put into sterile sampling bags and were frozen in the laboratory for DNA extraction. The seawater was filtered through a 0.22 μm filter membrane, and the membrane was stored at −20 °C for DNA extraction.

### 2.2. DNA Extraction and Sequencing

Using a commercial extraction kit (DNeasy PowerWater Kit), DNA was extracted according to the kit’s procedure. After extraction, DNA concentration was quantified using a microspectrophotometer (Thermo Fisher Scientific, Waltham, MA, USA), with an OD260 nm/280 nm ratio of 1.7 to 2.0 for all samples. The DNA was then amplified by PCR with the universal bacterial primers 515F(5′-GTTTCGGTGCCAGCMGCCGCGGTAA-3′) and 806R(5′-GCCAATGGACTACHVGGGTWTCTAAT-3′) [11], with the following procedure: 95 °C for 10 min, 30 cycles (95 °C for 30 s, 50 °C for 30 s, and 72 °C for 60 s) and 72 °C for 10 min. Finally, the PCR products were purified using commercial kits and subjected to high-throughput sequencing on the Illumina Hiseq 2500 platform (Novogene, Beijing, China) after library construction. The average sequencing data for each library was 3–4 M raw reads.

A high quality of DNA (PET and PS biofilms) was also subjected to metagenomic library construction and then sequenced on the Illumina Hiseq 2500 platform (Novogene, China).

### 2.3. Data Processing

#### 2.3.1. 16S rRNA Sequencing Data Analysis

The obtained forward and reverse reads were sequence spliced by mothur [12], and the primers and barcode base parts in the sequence were removed to prevent any impact on subsequent data analyses. Secondly, redundancy and chimera are removed based on Usearch [13], and OTUs are aggregated with a similarity of 97%. Via the EzBioCloud 16S rRNA gene database, ezbiocloud_full_align.fasta [14] performs species annotation on the obtained OTU sequence and then compares the obtained fasta sequence with the original reads to generate an OTU table.

#### 2.3.2. Metagenomic Data Analysis

The original reads were assembled by megahi [15], and the assembled contigs were binned based on three methods, Maxbin, metaBAT, and metaWRAP, to obtain MAGs (metagenome-assembled genomes) with different degrees of completeness and contamination [16]. Then MAGs with better quality were generated based on integrity and contamination by metawrap binrefinement, and the obtained genomes were species annotated by the GTDB (Genome Taxonomy Database) database [17,18,19]. Functional annotation was performed using the eggNOG database and KEGG database.

### 2.4. Data Analysis

Various statistical analyses were performed using the R software version 4.1.0 and the STAMP software version 2.1.3. The alpha diversity of the samples was calculated using the “vegan” R package, including Shannon, Simpson, Chao, and Ace indices. Upset plots were drawn using the UpSetR package. Bacterial community clustering between samples was visualized using the Principal Coordinate Analysis (PCoA). The “vegan” R package was used to determine the significance of clustering between samples. The “ggplot2” R package was used for plotting. A *p*-value of 0.05 was set as the significance level for all analyses. Ecological function was predicted for OTUs using FAPROTAX [20] and PICRUSt2 [21].

## 3. Results

### 3.1. Microbial Community Richness and Diversity

The Shannon and Chao1 indices showed the highest bacterial diversity on the surface of the PET samples, followed by Water, and finally, the PS samples (Table 1).

**Figure 1:**
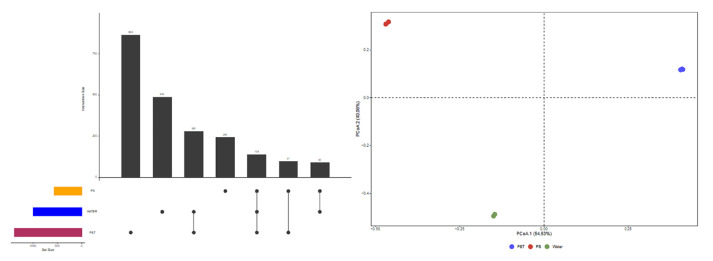
Upset and PCoA analysis.

The sequences obtained in the three types of samples were categorized into a total of 2200 bacterial OTUs. Upset plots showed 864 OTUs unique to PET, 243 to PS, and 488 to Water, which accounted for 62.65%, 42.78%, and 49.00% of the total number of their respective OTUs, respectively. Meanwhile, the PCoA analysis based on the Bray–Curtis distance (Figure 1) along with an ANOSIM analysis (R = 1, *p* = 0.015) showed significant differences in the community composition among the three sample types.

### 3.2. Microbial Community Structure Analysis

**Figure 2:**
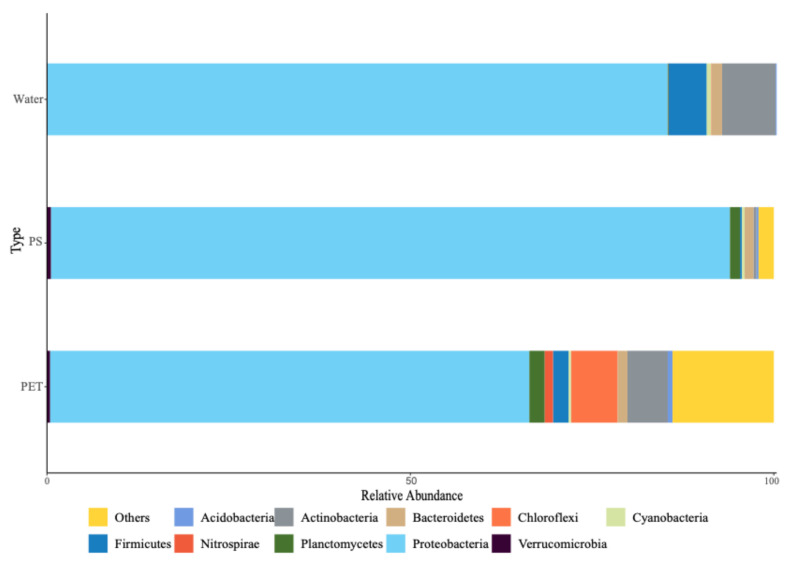
Bacterial stacking diagram at the phylum level.

A total of 26 phyla, 69 orders, 144 orders, 310 families, 771 genera, and 1369 species of bacteria were detected in this study, and the abundance of each species varied significantly among the samples. At the phylum level (Figure 2), the dominant phyla on the biofilm of PS were Proteobacteria (93.43%), Planktomycetes (1.38%), and Bacteroidetes (1.21%). The bacteria on the biofilm of PET were dominated by Proteobacteria (65.95%), Chloroflexi (6.42%), Actinobacteria (5.54%), Firmicutes (2.16%), Planctomycetes (2.12%), Bacteroidetes (1.35%), and Nitrospirae (1.13%), while the water sample was dominated by Proteobacteria (85.33%), Actinobacteria (7.38%), Firmicutes (5.25%), and Bacteroidetes (1.46%). At the genus level, the PS surface bacteria were mainly composed of *Alcanivorax* (8.28%), *Oleibacter* (7.87%), and *Chromatiales_o*, HQ397072_g (15.09%), and the PET surface bacteria were mainly composed of *Ruegeria* (12.93%), uncultured bacteria (*Alysiosphaera_o*, AJ581348_g) (6.29%) and *Erythrobacter* (4.30%).

### 3.3. Ecological Function Prediction Based on 16S rRNA Gene

**Figure 3:**
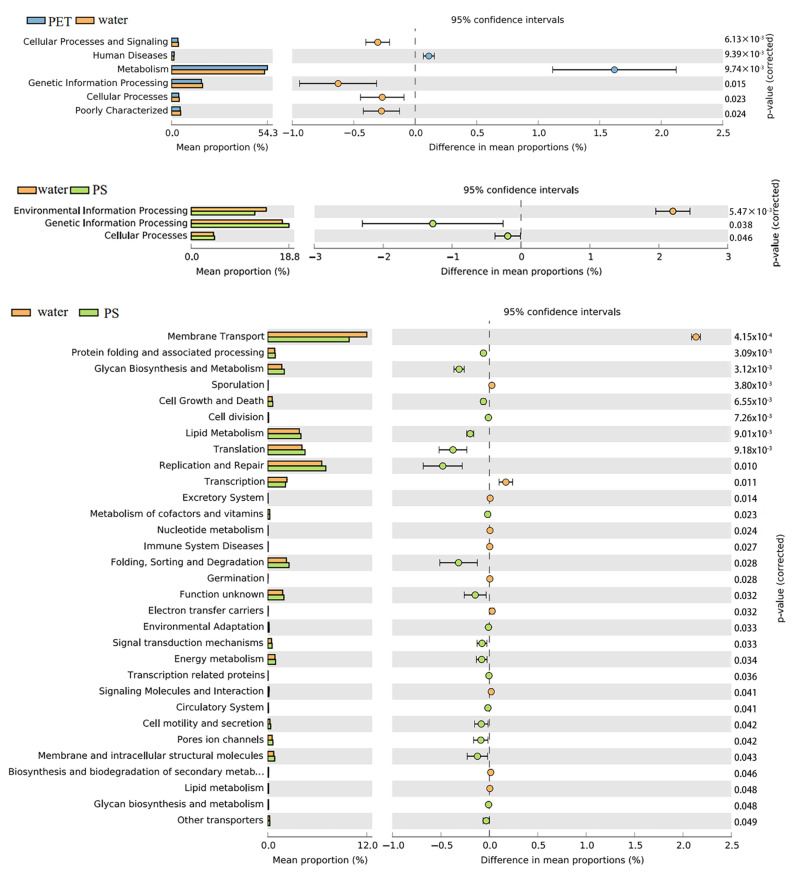

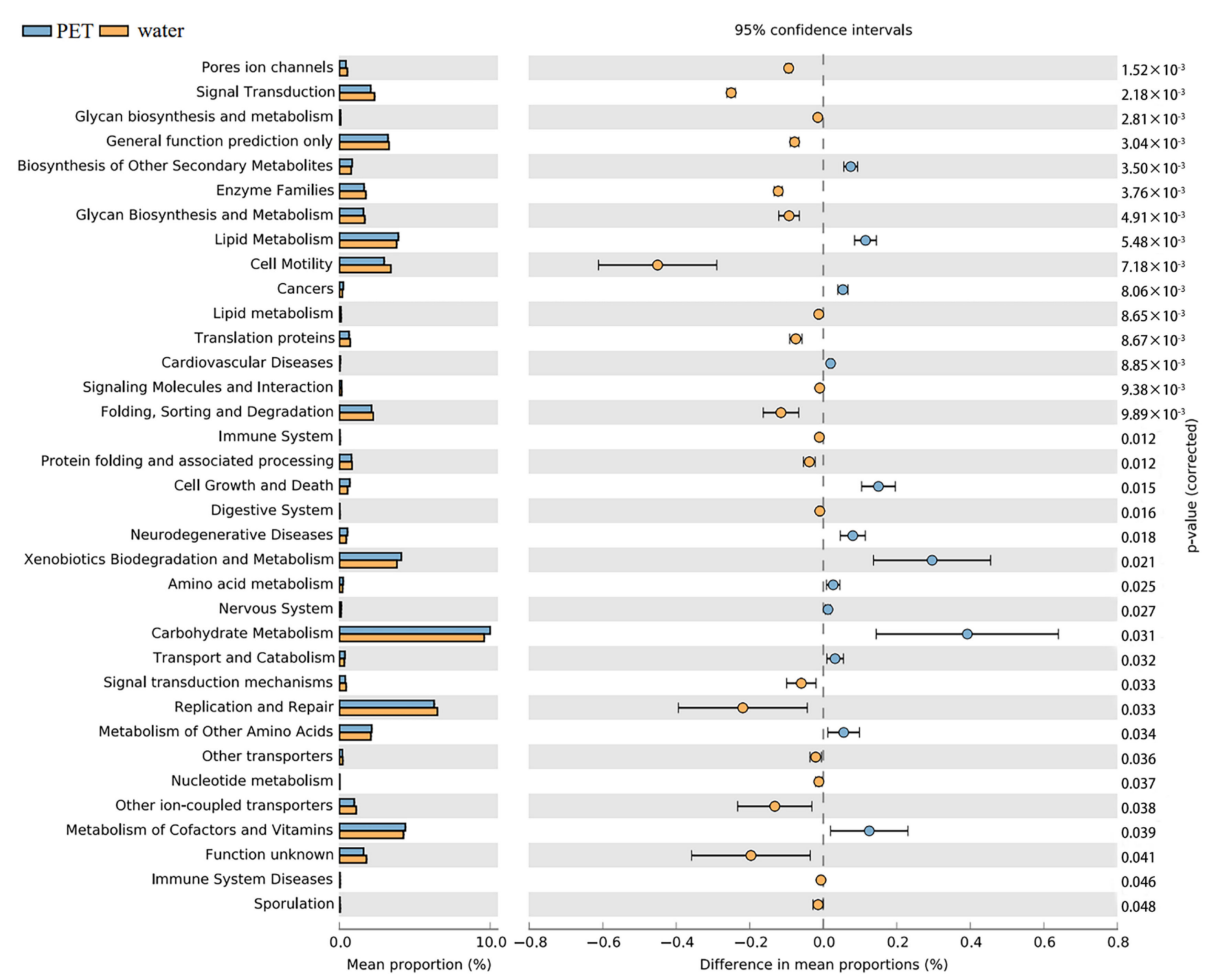
PICRUSt function prediction based on KEGG database.

Based on the KEGG and COG databases, PICRUSt2 was applied to predict the microbial functions of the three types of samples (Figure 3). The results show that three types of samples were involved in a total of six groups of biometabolic pathways, cellular processes, environmental information processing, genetic information processing, human diseases, metabolism, and organismal systems. Among them, metabolism was the dominant metabolic pathway, with abundance of 50.0–51.6%. The abundance of the functional genes of metabolism and human diseases in the PET samples was significantly higher than that in the water sample (*p* < 0.05). The abundance of genetic information processing and cellular processes in the PS samples were significantly higher than those in the water sample (*p* < 0.05), and the abundance of the remaining predicted genes in the first-level functional layer was not significantly different from those in the water sample. A further analysis of the predicted genes in the secondary functional layer showed that there were 41 subfunctions. Among them, the abundance of folding, sorting, and degradation genes in the PS biofilm was significantly higher than that in the water sample (*p* < 0.05), while the abundance of carbohydrate metabolism and xenobiotic biodegradation were significantly higher in the PET biofilm (*p* < 0.05) than in water.

**Figure 4:**
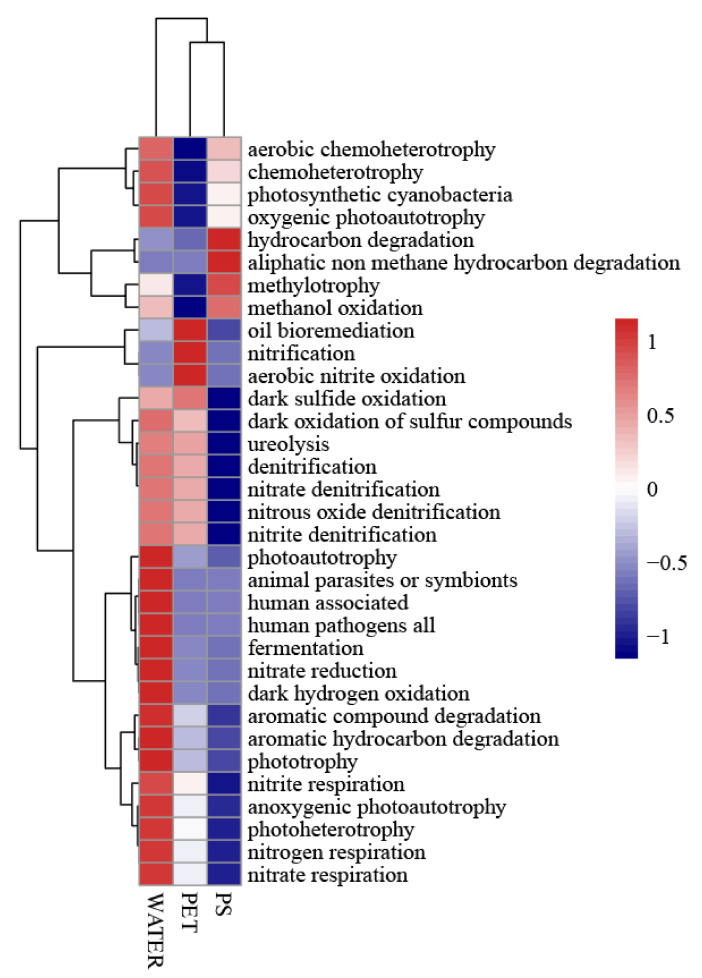
Ecological functions predicted by FAPROTAX.

The ecological functions were also predicted by applying FAPROTAX. FAPROTAX is capable of transforming bacterial community profiles that have been classified (typically in the form of OTU tables) into potential functional profiles. This conversion relies on taxonomic identifiers derived from annotated sequences of laboratory-cultured strains [20]. The process involves mapping prokaryotic clades (such as genera or species) to their respective metabolic or functional roles. As shown in Figure 4, there are significant differences in the bacterial community functions among the three types of samples. In the PS sample, the ecological functions with relatively high abundance are hydrocarbon degradation, aliphatic non-methane hydrocarbon degradation, methylotrophy, and methanol oxidation. Meanwhile, in the PET sample, besides oil bioremediation, the functions with high abundance are related to nitrogen and sulfur metabolism, including nitrification, aerobic nitrite oxidation, and dark sulfide oxidation.

### 3.4. Metagenomic Analysis

A total of 29 MAGs (with an integrity of >50% and a contamination of <10%) were generated from the PS sample, after assembling and binning, among which 13 were high-quality MAGs (with an integrity of >90% and a contamination of <10%). The 29 MAGs contained a total of 2 phyla, 3 classes, 11 orders, and 20 families, among which Pseudomonadales accounted for the largest proportion (48.3%). And a total of 341,521 genes were annotated at the contigs level. A total of 8 MAGs (>50% intact and <10% contigs) were generated from the assembled PET sample, containing 2 orders, 5 phyla, and 7 families. A total of 242,575 genes were annotated at the contigs level. In addition, two species of the genus *Alcanivorax* were found in the PS sample, *Alcanivorax borkumensis* and *Alcanivorax_s*. Among them, *Alcanivorax borkumensis* has been demonstrated to have the ability to degrade PE and petroleum in previous studies [22,23].

**Figure 5:**
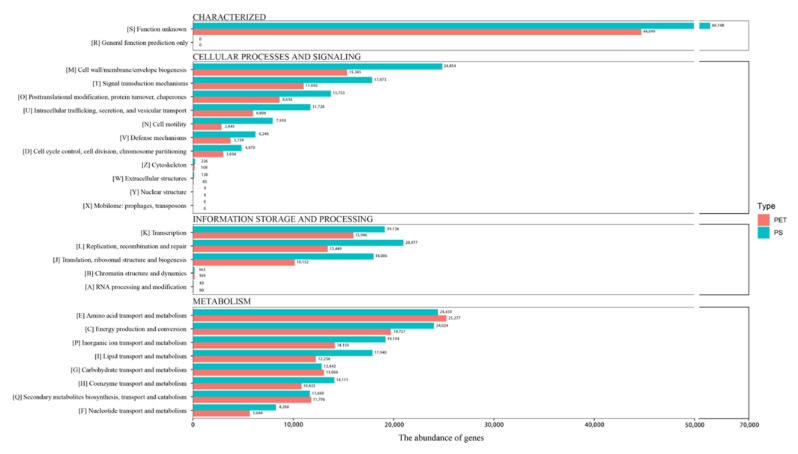
COG functional gene classification.

Based on the prediction results of the metagenome, the microbial functional genes on the surfaces of the PET and PS samples were annotated using the COG database. As shown in the Figure 5, for the PS sample, the top five COG categories with the highest enrichment are M (cell wall/membrane/envelope biogenesis) (7.21%); E (amino acid transport and metabolism) (7.09%); C (energy production and con-version) (6.97%); L (replication, recombination, and repair) (6.09%); and P (inorganic ion transport and metabolism) (5.57%), while for the PET sample, the order is E (amino acid transport and metabolism) (10.18%), C (energy production and conversion) (7.95%), K (transcription) (6.44%), M (cell wall/membrane/envelope biogenesis) (6.19%), and P (inorganic ion transport and metabolism) (5.70%).

**Figure 6:**
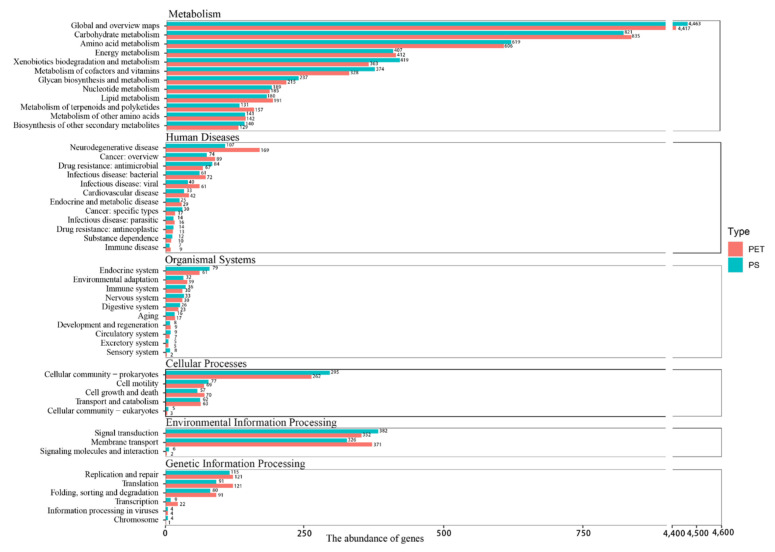
KEGG functional gene classification.

The KEGG annotation (Figure 6) results are similar to the COG annotation results, wherein both the PS and PET samples had a high abundance of metabolism-related genes, and the PS-gene abundance was higher than the PET-gene abundance. However, in human diseases, environmental information processing and genetic information processing, the abundance of PET sample genes was higher than that of the PS sample.

Based on the annotation results of contig and a previous study [24], a total of nine degradation genes related to PET or PS were annotated in this study (Table 2), including *MHETase*, *PETase*, *tphA2*, *tphA3*, *pcaG*, and *pcaH* for PET degradation and *styA*, *feaB*, and *paak* for PS degradation, among which *paak* had the highest abundance, accounting for 55.9% of the total abundance of the nine genes, while the second highest abundance was *pcaH* (21.5%) and the lowest abundance was *tphA3* (0.3%). The results of this study reveal that the high abundance of *paak* genes was present on the surface of PET, while *PETase* and *pcaH* were also present on the surface of PS.

**Figure 7:**
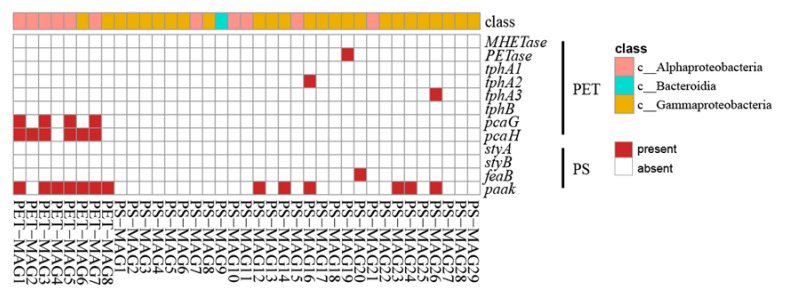
Statistical results of degradation genes based on MAGs.

The results based on MAGs (Figure 7) indicate that far fewer degradation genes were annotated in the respective MAGs than those annotated at the contig level, in which some genes were absent from the MAGs results. This may be due to the low integrity of the bacterial genome. Among the annotated degradation genes, *PETase*, *tphA2*, *tphA3*, and *feaB* appeared only in an individual genome, while *pcaG*, *pcaH*, and *paak* appeared in multiple genomes. Of these, *paak* occurs in both Alphaproteobacteria and Gammaproteobacteria, while *pcaG* and *pcaH* are mostly in Alphaproteobacteria.

## 4. Discussion

### 4.1. Microbial Community Structure on Plastic Debris

The microbial community of the plastic debris biofilms differed from the water sample. Bacterial richness and diversity in the PET biofilm were higher than the water sample, while for the PS biofilm, bacterial richness and diversity were lower than the water sample. Frère et al. [25] demonstrated that the species richness and diversity on the surface of plastics were higher than that of ambient samples, which is consistent with the PET results. It was also shown that the exposure time affected the species richness and diversity on the surface of plastics, which ultimately made it lower than that of ambient samples [26]. This is consistent with the PS results. The differences in microbial communities among different plastics may be due to the different materials on the one hand and the exposure time and aging degree of plastics in the environment on the other hand.

Previous studies have shown that the plastisphere is dominated by Proteobacteria, Bacteroidetes, and Cyanobacteria, with a significant presence of hydrocarbon-degrading bacteria, such as *Alcanivorax* and *Marinobacter* [8,22]. In our study, Proteobacteria is the most abundant phylum (85.33%, 65.95%, and 93.43%) among all samples and is the main bacterial constituent on the biofilm of PET and PS. This result is in agreement with previous studies [27,28]. This further proves the dominant position of Proteobacteria in the natural environment. At the genus level, the dominant genera *Alcanivorax, Oleibacter* and *Erythrobacter*, which belong to α-proteobacteria and γ-proteobacteria, were significantly correlated with PET and PS [5,29,30] and petroleum hydrocarbon degradation [28,31]. The genus *Alcanivorx* has also been shown to be effective in LDPE degradation [22]. In this study, the sampling site was close to a water catering site, and the ship traffic and catering wastewater may provide favorable conditions for the growth of microorganisms with hydrocarbon-degradation abilities. In previous studies [4,32,33], potential pathogens such as *Vibrio* and *Pseudoalteromonas* were often associated with plastics. Our results also show these pathogens did colonize on the biofilm of PS and PET in small amounts but were enriched in the water sample. The percentage of *Vibrio* spp. in the water sample was 11.35%, *Pseudoalteromonas* was 10.59%, and *Toxoplasma gondii* was 5.01%. The species in genus *Pseudomonas* are known as PS- and PET-degrading bacteria [34,35]. *Pseudomonas* spp. were investigated to be presented in small amounts on the biofilm of both PS (0.14%) and PET (0.08%) samples, suggesting that even if microorganisms with a degradation ability exist on the surface of plastics in natural environments, they are not ecologically predominant and therefore cannot effectively degrade the samples, so the screening of plastic-degrading bacteria is of great significance for the application of dealing with plastic pollution.

### 4.2. Metagenomic Sequencing Provided Comprehensive Information about Plastic Degradation Potential

Metagenomic sequencing can profile the taxonomic composition and the functional potential of microbial communities. After assembling and binning, two species of the genus *Alcanivorax* were found on the PS sample. This result is consistent with the high-throughput sequencing based on the 16S rRNA gene. Moreover, the assembly results in this study also show the presence of alkane degradation genes, such as *AlkB*, *rubA*, and *rubB* in binned genomes, but whether *Alcanivorax* is degradable to PS remains to be further investigated. Functional annotations based on the eggNOG database and KEGG database were similar. The number of functional genes on the PS biofilm was higher than that of the PET biofilm in information storage and processing, metabolism, and cellular processes and signaling. This study annotated a total of nine degradation genes related to PET or PS, strongly suggesting that the surface of plastic debris harbors a significant amount of these degradation genes. Moreover, our analysis reveals a considerable presence of the PS degradation gene, *paak*, on the surface of PET, alongside the PET degradation genes, *PETase* and *pcaH*, found on the surface of PS. This observation might suggest a potential interconnection between the degradation genes of specific plastics.

## 5. Conclusions

Plastic pollution has become a significant problem worldwide, as plastic debris act as carriers for organic pollutants and microorganisms. This study showed that the bacterial community structure on plastic debris biofilms is less related to the environment (water) and more related to the type of plastic. The phylum Proteobacteria was the most dominant component of the water and biofilm bacterial communities. Both PS and PET biofilms were enriched with a number of hydrocarbon (petroleum, microplastics, etc.)-degrading genes, suggests the presence of a large number of degradation genes in the natural environment.

## Figures and Tables

**Table 1:** Alpha diversity index.

Sample	Shannon	Simpson	Chao	Ace
PET	5.821	0.987	1749.185	1896.405
Water	5.227	0.980	1149.878	1248.644
PS	4.612	0.958	597	589.058

**Table 2:** Functional genes for plastic biodegradation.

Target Plastic	Gene	PS	PET
PET	*MHETase*	1	1
	*PETase*	19	2
	*tphA1*	-	-
	*tphA2*	3	1
	*tphA3*	1	-
	*tphB*	-	-
PS	*pcaG*	6	15
	*pcaH*	17	45
	*styA*	2	-
	*styB*	-	-
	*feaB*	6	8
	*paak*	57	104

## Data Availability

The original contributions presented in the study are included in the article, further inquiries can be directed to the corresponding author. The raw sequence data for 16S rRNA and metagenome were deposited under NCBI BioProject Accession Nos. PRJNA1130660 and PRJNA1129439.
